# Aberrant activation of bone marrow Ly6C ^high^ monocytes in diabetic mice contributes to impaired glucose tolerance

**DOI:** 10.1371/journal.pone.0229401

**Published:** 2020-02-25

**Authors:** Yosuke Ikeda, Noriyuki Sonoda, Battsetseg Bachuluun, Shinichiro Kimura, Yoshihiro Ogawa, Toyoshi Inoguchi

**Affiliations:** 1 Department of Medicine and Bioregulatory Science, Graduate School of Medical Sciences, Kyushu University, Fukuoka, Japan; 2 Department of Molecular Medicine and Metabolism, Research Institute of Environmental Medicine, Nagoya University, Aichi, Japan; 3 Department of Molecular and Cellular Metabolism, Graduate School of Medical and Dental Sciences, Tokyo Medical and Dental University, Tokyo, Japan; 4 CREST, Japan Agency for Medical Research and Development, Tokyo, Japan; 5 Fukuoka City Health Promotion Support Center, Fukuoka, Japan; Albert Einstein College of Medicine, UNITED STATES

## Abstract

Accumulating evidence indicates that diabetes and obesity are associated with chronic low-grade inflammation and multiple organ failure. Tissue-infiltrated inflammatory M1 macrophages are aberrantly activated in these conditions and contribute to hyperglycemia and insulin resistance. However, it is unclear at which stage these cells become aberrantly activated: as precursor monocytes in the bone marrow or as differentiated macrophages in tissues. We examined the abundance, activation state, and function of bone marrow-derived Ly6C^high^ monocytes in mice with diabetes and/or obesity. Ly6C^high^ monocytes were FACS-purified from six groups of male mice consisting of type 2 diabetes model *db/db* mice, streptozotocin (STZ) induced insulin depletion mice, high fat diet (HFD) induced obesity mice and each control mice. Ly6C^high^ monocytes were then analyzed for the expression of inflammation markers by qRT-PCR. In addition, bone marrow-derived Ly6C^high^ monocytes from *db/+* and *db/db* mice were fluorescently labeled and injected into groups of *db/db* recipient mice. Cell trafficking to tissues and levels of markers were examined in the recipient mice. The expression of many inflammation-related genes was significantly increased in Ly6C^high^ monocytes from *db/db* mice, compared with the control. Bone marrow-derived Ly6C^high^ monocytes isolated from *db/db* mice, but not from *db/+* mice, displayed prominent infiltration into peripheral tissues at 1 week after transfer into *db/db* mice. The recipients of *db/db* Ly6C^high^ monocytes also exhibited significantly increased serum glucose levels and worsening tolerance compared with mice receiving *db/+* Ly6C^high^ monocytes. These novel observations suggest that activated Ly6C^high^ monocytes may contribute to the glucose intolerance observed in diabetes.

## Introduction

Chronic low-grade inflammation is an important contributor to multiple organ failure in patients with diabetes and obesity [1, 2]. The prevalence of metabolic syndrome including obesity and diabetes continues to increase. We have previously reported that inflammatory and oxidative stress mediators, including reactive oxygen species produced via protein kinase C (PKC)-dependent activation of NAD(P)H oxidase, contributes to the development of atherosclerotic complications in patients with diabetes and metabolic syndrome [3, 4]. Inflammatory M1 macrophages are thought to play important roles in diabetes and obesity through infiltration into adipose tissue and production of reactive oxygen species and inflammatory mediators, which cause chronic local inflammation [5, 6] and dysregulation of adipocytokines [7]. Tissue macrophages originate from precursor monocytes produced in the bone marrow, which circulate through the blood until they migrate into and differentiate within tissues. In patients with diabetes and obesity, it is not known whether the precursor monocytes are already activated before arrival at the tissue or become activated upon differentiation. Similarly, the extent to which bone marrow-derived monocytes may contribute to the chronic inflammation observed in obesity and diabetes remains unclear. Pro-inflammatory (CD14+ CD16+) monocytes are more abundant in the peripheral blood of patients with type 2 diabetes compared with normal subjects [8], suggesting that this may indeed be the case.

In mice, multiple phenotypic and functionally distinct monocyte subsets have been described, including the so-called “inflammatory” (Ly6C^high^) and “patrolling” (Ly6C^low^) subsets [9]. However, there have been no previous studies of potential changes in the abundance and function of bone marrow-derived monocytes in obese or diabetic animals. To address this, we employed three mouse models of diabetes and obesity and investigated (i) disease-related alterations in the number and inflammatory status of bone marrow-derived monocytes and (ii) the potential contribution of Ly6C^high^ monocytes to tissue inflammation and dysregulation of glucose homeostasis. We confirmed that diabetic bone marrow monocytes had abnormal activation and had an effect on chronic inflammation.

## Materials and methods

### Animals and diet

Seven-week-old male C57BL/KsJ *db/db* mice, an experimental model of type 2 diabetes, lean *db/+* littermates, and wild-type C57BL/6J mice were purchased from Oriental Yeast (Tokyo, Japan) and housed for 1 week to allow acclimation before use in experiments. Mice were maintained under standard pathogen-free conditions with free access to water and normal laboratory chow.

Diabetes was induced in 8-week-old C57BL/6J mice by i.p. injection of streptozotocin (STZ, 50 mg/kg body weight) (Sigma-Aldrich, St. Louis, MO, USA) in 0.1 mol/l citrate buffer (pH 4.5) once daily for 5 consecutive days (n = 11). Diabetes was confirmed by the presence of hyperglycemia (plasma glucose levels >300 mg/dl). Mice injected with citrate buffer alone served as non-diabetic controls (n = 11). In addition, groups of 8-week-old C57BL/6J mice (n = 11) were fed normal control diet (CD) or a high-fat diet (HFD) for an additional 8 weeks. The CD contained 11.5, 70.3, and 18.2% calories from fat, carbohydrate, and protein, respectively, and a total of 3.53 kcal/g. The HFD contained 62.2, 19.6, and 18.2% calories from fat, carbohydrate, and protein, respectively, and a total of 5.06 kcal/g. The mice used in our experiment were male only. All mice were anesthetized with isofurane and bone marrow was harvested by flushing the femurs and tibia, and then they were killed by exsanguination. All methods were performed in accordance with the relevant guidelines and regulations. Every efort was made to minimize the number of animals used and their suffering. All animal protocols were reviewed and approved by the Committee on the Ethics of Animal Experiments, Graduate School of Medical Sciences, Kyushu University (Protocol Number: A19-046-0).

### Monocyte isolation and labeling

Bone marrow was harvested by flushing the femurs and tibia with RoboSep Buffer (Stemcell Tech, Vancouver, BC, Canada) using a syringe with a 27-gauge needle. Cell clumps were removed by passing the cell suspension through a 70-μm mesh nylon strainer and the cells were centrifuged at 300 × g for 6 min. The pelleted cells were resuspended at 1 × 10^8^ cells/ml, and monocytes were enriched with an EasySep Mouse Monocyte Enrichment kit according to the manufacturer’s instructions.

Isolated monocytes (5 × 10^5^ to 1 × 10^6^) were labeled with PKH26 using a PKH26 Labeling kit (Sigma-Aldrich, St. Louis, MO, USA) according to the manufacturer’s instructions. PKH26 fluorescence (a yellow-orange fluorescent dye with long aliphatic tails) technology provides stable incorporation into lipid regions of cell membranes and has been found to be useful for in vitro and in vivo cell tracking applications in a wide variety of systems. In brief, cells were washed once in serum-free RPMI-1640 medium, resuspended in 2 mL kit diluent solution C, mixed with PKH26 at 2 × 10^−3^ mol/L in diluent C, and incubated for 10 min at room temperature in the dark. An equal volume of medium containing 10% FBS was added, and the cells were centrifuged, washed once, and resuspended in serum-containing medium for further analysis.

### FACS sorting

Purified monocytes were incubated with Fc blocker (BD Biosciences, San Jose, CA, USA) at 4°C for 5 min, counted, and incubated for an additional 30 min in the dark on ice with fluorescently labeled antibodies against mouse F4/80, CD11b, CD115, Ly6G, and Ly6C. All antibodies were purchased from Biolegend (San Diego, CA, USA). Cell sorting was performed using a FACSAriaⅡ flow cytometer (BD Biosciences, San Jose, CA, USA).

### Adoptive transfer of monocytes to db/db mice

Ly6C^high^ monocytes from *db/db* or *db/+* mice were label with PKH26, resuspended in PBS at 4 × 10^5^ viable cells/0.2 ml, and injected into the jugular vein of groups of recipient *db/db* mice. One week later, one set of mice were analyzed for tissue infiltration. The liver and kidneys were collected, cryopreserved in OCT compound, cut into 10-μm-thick sections on a Leica CM1950 cryostat (Leica Biosystems, Nussloch, Germany), and mounted on slides. Adipose tissue was collected, cryopreserved in super cryoembedding medium (SCEM-(L1)), cut into 50-μm-thick sections, and mounted. PKH26+ cells were detected by fluorescence microscopy (model BZ-X700; Keyence, Osaka, Japan) at excitation and emission wavelengths of 551 and 567 nm, respectively. For quantification, the number of cells in 16 high-power fields per sample were counted.

Body weight, IPGTT, IPITT, and levels of blood glucose, serum fructosamine, urinary glucose, urinary albumin, and urinary 8-hdroxy-2′-deoxyguanosine (8-OHdG), were monitored in additional groups of *db/db* recipient mice at various times over 4 weeks after cell transfer, as described below.

### RNA extraction and quantitative RT-PCR

RNA was extracted from FACS-sorted monocytes using a RNeasy Plus Mini Kit (250) (Qiagen, Chatsworth, CA, USA) and reverse-transcribed using QuantiTect Reverse Transcription Kit (Qiagen). PCR was performed on a Chromo4 real-time PCR system (Bio-Rad, Hercules, CA, USA) with GoTaq Green Master Mix (Promega, Woods Hollow, WI, USA), as described previously [10–12]. The mRNA expression of each gene was normalized to the expression of the reference gene *β-actin*. The specificity of PCR amplification was confirmed by melting curve analysis and agarose gel electrophoresis.

### Luseogliflozin treatment

The SGLT2 inhibitor luseogliflozin (TS-071: (1S)-1,5-anhydro-1-[5-(4-ethoxybenzyl)-2-methoxy-4-methylphenyl1]-1-thio-d-glucitol), was synthesized and kindly provided by Taisho Pharmaceutical Co., Ltd. (Tokyo, Japan). Luseogliflozin was administered by mixing it with the chow at a concentration of 0.1%. This dose was selected to ensure normalization of mild hyperglycemia regardless of variations in daily food consumption in individual mice. Two groups each of 8-week-old *db/db* and *db/+* mice were assigned to receive food mixed with luseogliflozin or a normal diet for 12 weeks. Body weights and blood glucose levels were monitored every 2 weeks. After 12 weeks, Ly6C^high^ monocytes were isolated from bone marrow samples and analyzed by RT-PCR as described above.

### IPGTT and IPITT

Glucose and insulin tolerance tests were performed in *db/db* mice 4 weeks after transfer of PKH26-labeleled Ly6C^high^ monocytes from *db/db* or *db/+* mice. Mice were fasted for 16 h and then injected i.p. with glucose at 1.0 g/kg body weight. Plasma glucose was determined as described previously [12]. The AUC was calculated by the trapezoidal rule. For the IPITT, mice were injected i.p. with 0.5 U/kg of human biosynthetic insulin (Novo Nordisk, Bagsvaerd, Denmark) 4 weeks after cell transfer, and blood glucose levels were measured as described previously [12].

### Urinalysis

Four weeks after monocyte transfer to *db/db* mice, the mice were placed in metabolic cages and urine was collected for 24 h. The urine was mixed, centrifuged at 7500 × g for 5 min, purged of air with a stream of nitrogen to prevent formation of oxidation products, and then stored at −80°C until analysis. 8-OHdG levels were measured using an 8-OHdG Check ELISA kit (Japan Institute for the Control of Aging, Fukuroi, Japan) as previously described [13]; albumin concentrations were measured using a Mouse Albumin ELISA KIT (AKRAL-121, Shibayagi, Gunma, Japan); and creatinine concentrations were measured using an automated analyzer. 8-OHdG and albumin levels are expressed relative to the urinary creatinine level.

### Statistical analysis

Data are expressed as the means ± standard error (SEM). Student’s t-test was used when two groups were compared. Multiple comparisons among the groups were conducted by one-way ANOVA with Fisher’s protected least significant difference test. A value of P < 0.05 was considered statistically significant.

## Results

### Body weight and glucose levels in diabetic mice

**Fig 1:**
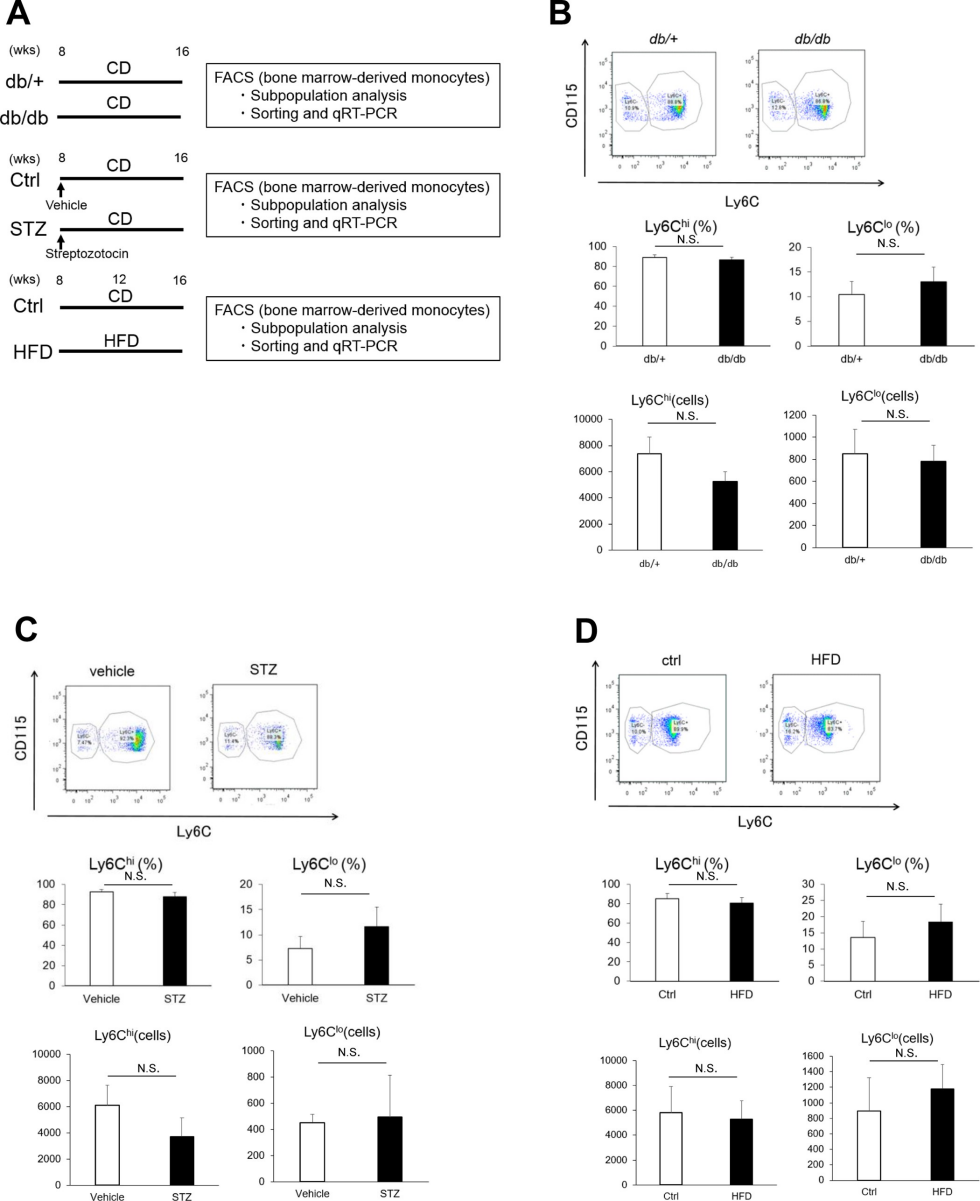
Experimental protocol and flow cytometry results. (A) Schematic showing experimental protocol. Upper row: Eight-week-old *db/+* and *db/db* mice were fed normal control diet (CD) for an additional 8 weeks. Middle row: Eight-week-old C57BL/6 mice were injected with citrate buffer (vehicle) or streptozotocin (50 mg/kg) once daily for 5 days and fed CD for an additional 8 weeks. Lower row: Eight-week-old C57BL/6 mice were fed CD or a high-fat diet (HFD) for an additional 8 weeks. Mice were sacrificed and bone marrow cells were analyzed by flow cytometry for the ratio of Ly6C^high^ to Ly6C^low^ monocytes. Ly6C^high^ monocytes were sorted and analyzed by quantitative RT-PCR. (B-D) Representative flow cytometry dot plots (upper panels) and quantification (lower panels) of CD115+ Ly6C^high^ and Ly6C^low^ in monocytes obtained from the bone marrow of (B) *db/db* mice and *db/+*, (C) vehicle- and STZ-treated mice, and (D) Ctrl- or HFD-fed mice. Data are the expressed as the means ± SEM. N = 11 mice/group. Ctrl, control diet; HFD, high-fat diet; STZ, streptozotocin. N.S., not significant. (unpaired t-test).

**Fig 2:**
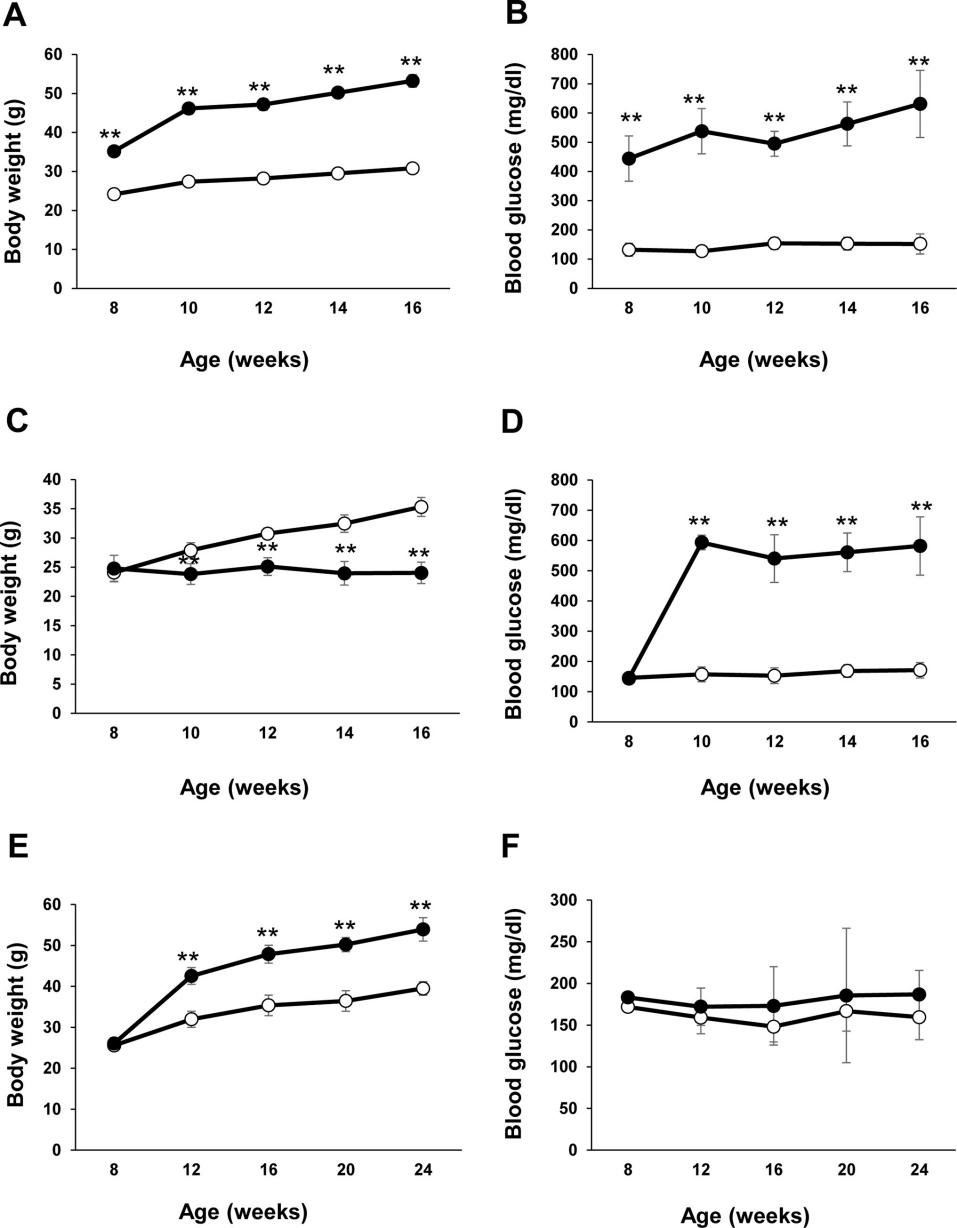
Body weight and glucose levels. The body weights and blood glucose levels of 8 to 16 weeks-old *db/db* (black circle), *db/+* (white circle) mice (A-B), C57BL/6 mice administered STZ (black circle) or vehicle (white circle) (C-D), and C57BL/6 mice fed CD (white circle) or a HFD (black circle) (E-F). Data are the expressed as the means ± SEM. N = 11 mice/group. **p<0.01. Ctrl, control diet; HFD, high-fat diet; STZ, streptozotocin. (unpaired t-test).

To assess the development of diabetes in each of the three mouse models studied here, we examined the body weights and blood glucose levels of 8-week-old *db/db*, *db/+* mice, C57BL/6 mice administered STZ or vehicle, and C57BL/6 mice fed CD or a HFD until they were 16 weeks of age (Figs 1 and 2). As shown in Fig 2, the postprandial blood glucose levels were significantly higher in *db/db* mice and STZ-treated mice than in the age- and sex-matched control mice, starting at the first week of analysis (Fig 2B and 2D), whereas the HFD mice showed a trend towards higher glucose levels than CD-fed mice, but the difference was not significant (Fig 2). In contrast, although the body weights of *db/db* and HFD-fed mice were significantly higher than the control mice (Fig 2A and 2E), the STZ-treated mice had significantly lower body weights than the vehicle-treated mice (Fig 2C). Insulin secretion is decreased by destroying β cells in the pancreas when mice were treated streptozotocin. The condition that glucose cannot be taken into the tissue becomes chronic, a lack of energy in the tissue and weight loss may occur because of getting energy from fat and muscle.

### Analysis of Ly6C^high^ monocyte subpopulations and expression of oxidative stress- and other inflammation-associated genes

**Fig 3:**
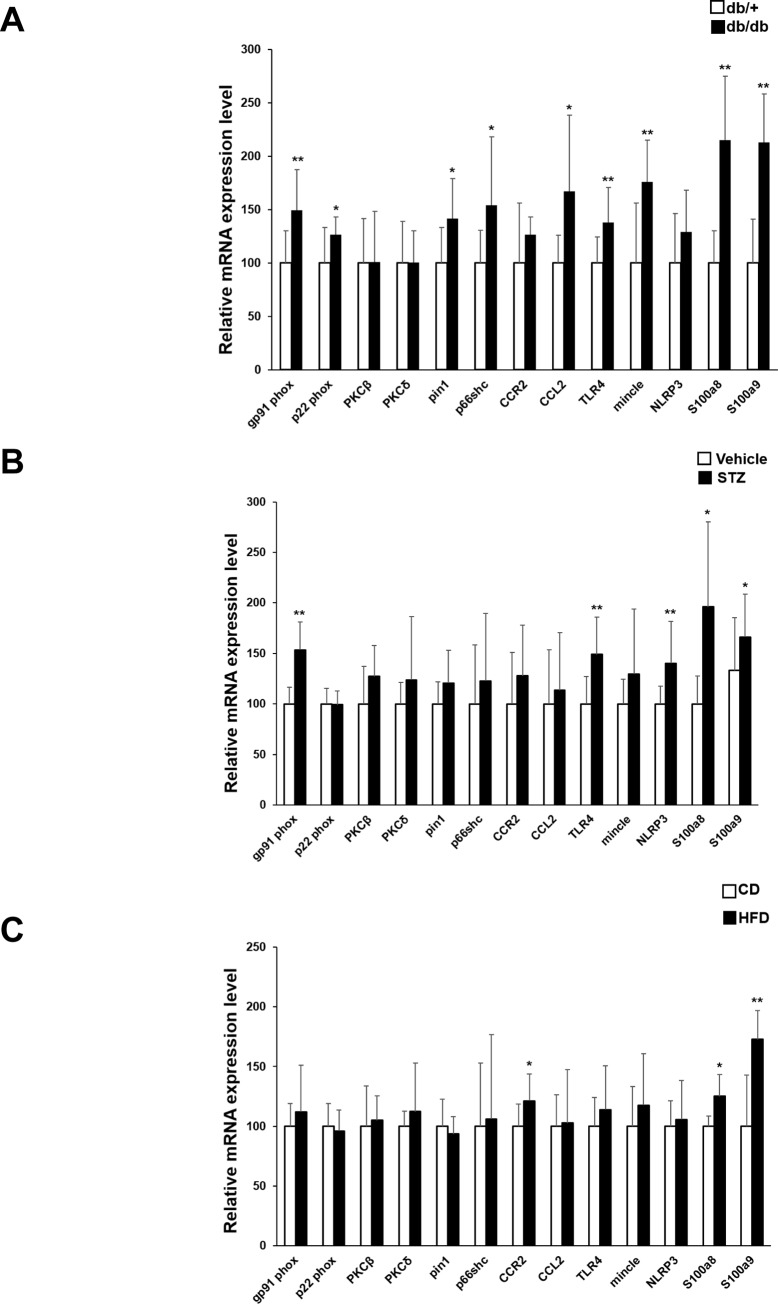
Comparison of expression of inflammation related gene marker of Ly6C^hi^ cells in pathological model. qRT-PCR analysis of expression of the indicated genes in monocytes from (A) *db/+* and *db/db* mice, (B) vehicle- and STZ-treated mice, and (C) CD- or HFD-fed mice. Data are expressed as the means ± SEM. N = 11 mice/group. *p<0.05, **p<0.01. CD, control diet; HFD, high-fat diet; STZ, streptozotocin. (unpaired t-test).

To determine whether pro-inflammatory (Ly6C^high^) monocytes were more abundant and/or displayed a more activated phenotype in diabetic and obese mice compared with control mice, we harvested bone marrow cells from *db/+* and *db/db* mice, vehicle- or STZ-treated mice, and CD- or HFD-fed mice at 16 weeks of age (Fig 1). Monocytes were identified as F4/80+Ly6G-CD11b+CD115+ cells. Ly6C^high^ and Ly6C^low^ monocytes were enumerated, and the latter subpopulation was purified by sorting for qRT-PCR analysis. Interestingly, the ratio of Ly6C^high^ to Ly6C^low^ monocytes and absolute numbers of Ly6C^high^ and Ly6C^low^ monocytes did not differ significantly between the three types of diabetic and/or obese mice and their relevant controls (~85% and 15%, respectively, Fig 1B–1D), indicating that induction of diabetes or obesity did not increase the proportion of pro-inflammatory monocytes. Therefore, we next compared the functional status of the Ly6C^high^ monocytes by analyzing expression of a panel of genes related to inflammation, including oxidative stress markers. As shown in Fig 3A, mRNA levels of the NADPH oxidase subunits *gp91phox* and *22phox*, peptidyl-prolyl cis-trans isomerase NIMA-interacting 1 (*Pin1*), *p66shc*, *CCL2*, *TLR4*, *mincle*, *S100a8*, and *S100a9* were significantly higher in Ly6C^high^ monocytes from *db/db* mice than the cells from *db/+* mice (Fig 3A). In addition, *gp91phox*, *TLR4*, *NLRP3*, *S100a8*, and *S100a9* were significantly higher in Ly6C^high^ monocytes from STZ-treated mice than in control mice, with modest increases in other inflammatory markers (Fig 3B). However, only *CCR2*, *S100a8*, and *S100a9* mRNA levels were elevated in Ly6C^high^ monocytes from HFD-fed compared with CD-fed mice (Fig 2C). Thus, Ly6C^high^ monocytes from diabetic or obese mice expressed significantly higher levels of oxidative stress and other inflammation markers than cells from the control mice, consistent with a more pro-inflammatory phenotype.

### Normalization of blood glucose does not suppress aberrant activation of Ly6C^high^ monocytes in diabetic mice

**Fig 4:**
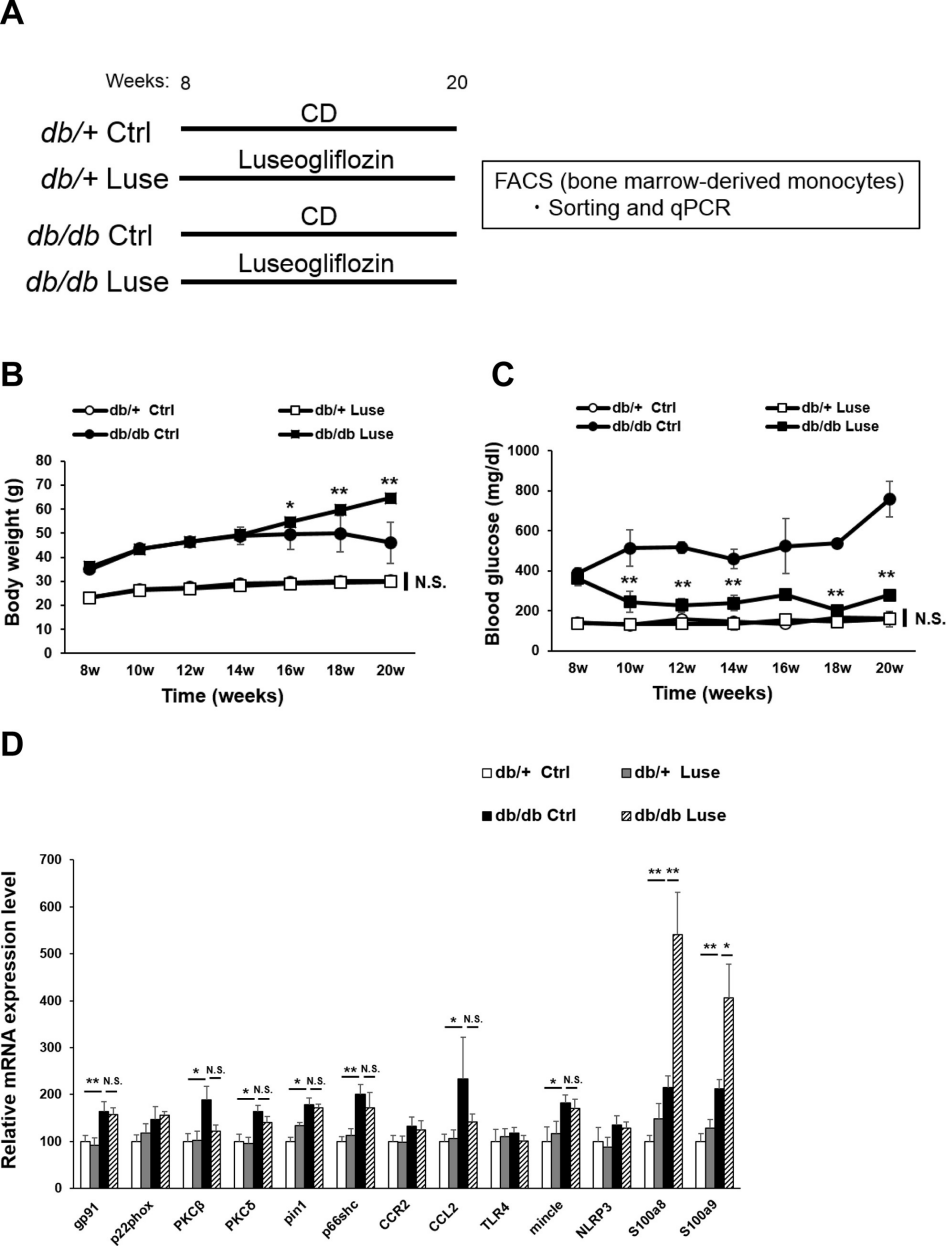
Gene expression change of monocytes using blood glucose lowering model. (A) Schematic showing experimental protocol. Eight-week-old *db/+* and *db/db* mice were fed CD or CD mixed with the SGLT2 inhibitor luseogliflozin for an additional 12 weeks. Mice were sacrificed and Ly6C^high^ monocytes were sorted from the bone marrow and analyzed. Body weight (B) and blood glucose levels (C) in *db/+* and *db/db* mice treated as described in (A). Results are presented as the means ± SEM. N = 6 mice/group. *p<0.05, **p<0.01. CD, control diet; Ctrl, control; Luse, luseogliflozin. (D) qRT-PCR analysis of expression of the indicated genes in monocytes from mice treated as described in 4A. Data are expressed as the means ± SEM. N = 6 mice/group, *p<0.05, **p<0.01. Luse, luseogliflozin; N.S., not significant. (ANOVA with Fisher’s PLSD).

To evaluate whether the elevated expression of inflammation-related markers in Ly6C^high^ monocytes of diabetic mice were related to high glucose levels, we treated one set of *db/db* and *db/+* mice with luseogliflozin, an inhibitor of sodium glucose transporter 2 (SGLT2), the major protein responsible for glucose resorption in the kidney. Luseogliflozin was administered for 12 weeks by mixing with food (Fig 4A). *db/+* mice showed no effects of luseogliflozin on body weights or blood glucose levels, as expected; however, luseogliflozin-treated *db/db* mice showed significantly reduced blood glucose levels at all time points examined, and their body weights were significantly elevated compared with the control mice after 8 weeks of treatment (Fig 4B and 4C). qRT-PCR analysis of bone marrow-derived Ly6C^high^ monocytes from these mice revealed that, as expected, basal mRNA levels of most inflammation-associated genes were significantly higher in cells from untreated control *db/db* mice than *db/+* mice (Fig 4D). However, with the exception of S100a8 and S100a9, luseogliflozin had no effect on the expression of inflammation-associated markers in the cells from *db/db* mice, although there was a small but insignificant trend towards reduced expression of some genes (Fig 4D). Gene expression profiles of Ly6Chigh monocytes from *db/+* vs *db/db* from two experiments show discrepancy (in particular, between Fig 3A and Fig 4D). This may be part of the reason that Ly6C^high^ cells were collected from different time points (~ 600 mg / dl blood glucose for 16 weeks vs ~ 800 mg / dl for 20 weeks).

### Infiltration of adoptively transferred Ly6C^high^ monocytes into liver, kidneys, and adipose tissue of db/db mice

**Fig 5:**
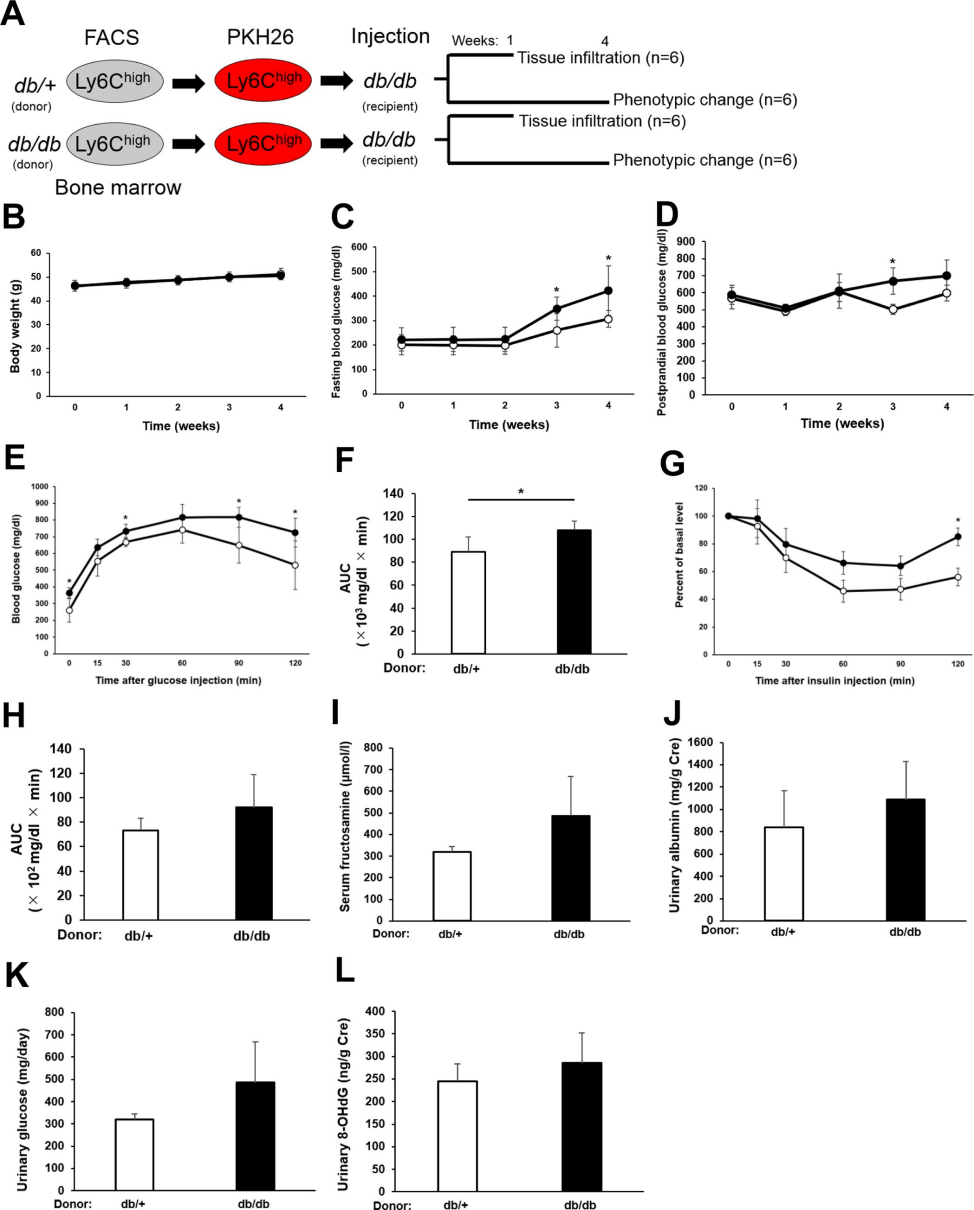
Cell trafficking examination. (A) Schematic showing experimental protocol. Ly6C^high^ monocytes were sorted from the bone marrow of *db/+* or *db/db* mice, labeled with PKH26, and injected i.v. into *db/db* mice. One group of mice was sacrificed after 1 week and analyzed for infiltration of PKH26+ cells in liver, kidneys, and adipose tissue. A second group of mice were tested for phenotypic changes at various times for 4 weeks after cell transfer. (B-D) Body weight (b), fasting blood glucose levels (c), and postprandial blood glucose levels (d) in *db/db* mice at the indicated times after transfer of *db/+* Ly6C^high^ monocytes (white circles) or *db/db* Ly6C^high^ monocytes (black circles) as described in 4(a). Results are expressed as the means ± SEM. N = 6 mice/group. *p<0.05. (unpaired t-test) (E and F) IPGTT of *db/db* mice at 2 weeks after transfer of *db/+* or *db/db* bone marrow-derived Ly6C^high^ monocytes as described in 4(A). (E) Blood glucose levels. (F) IPGTT AUC. Results are expressed as the means ± SEM. N = 6 mice/group, *p<0.05. (unpaired t-test) (G and H) IPITT of *db/db* mice at 4 weeks after transfer of *db/+* or *db/db* bone marrow-derived Lyc6^high^ monocytes as described in 4(A). (G) Blood glucose levels. (H) AUC. Results are expressed as the means ± SEM. N = 6 mice/group. *p<0.05. (unpaired t-test) (I-L) Serum fructosamine levels (I), urinary glucose levels (J), urinary albumin levels (K), and urinary 8-hydroxyl-2′-deoxyguanosine (8-OHdG) levels (L) in *db/db* mice at 4 weeks after injection of *db/+* or *db/db* Ly6C^high^ monocytes as described in 4(A). Results are expressed as the means ± SEM. N = 6 mice/group. *p<0.05. (unpaired t-test) Cre, creatinine.

**Fig 6:**
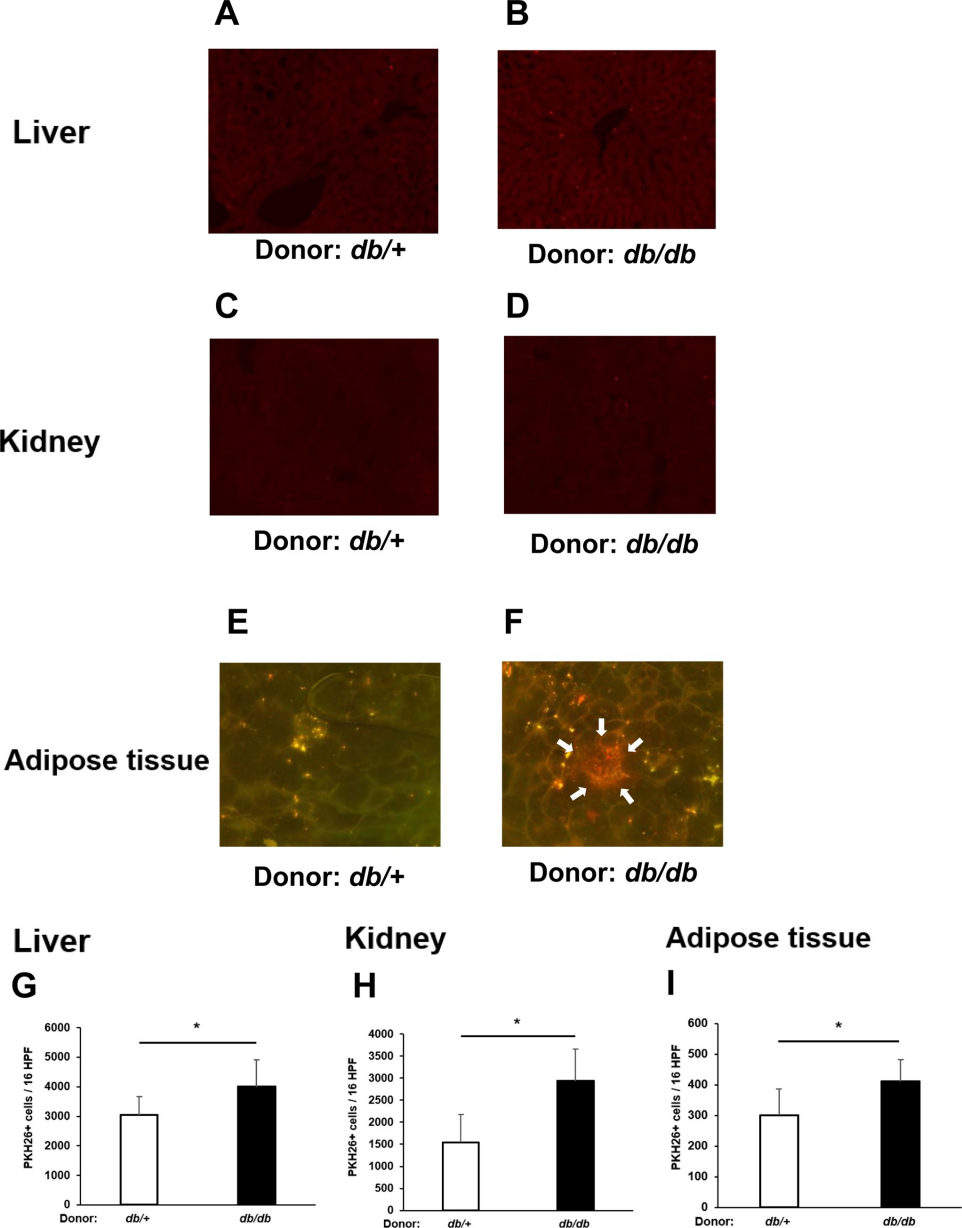
Cell trafficking examination. (A-F) Immunofluorescence microscopy of infiltration of PKH26-labeled *db/db*-derived or *db/+*-derived Ly6C^high^ monocytes in the liver (A, B), kidney (C, D), and adipose tissue (E, F) of recipient *db/db* mice, as described in Fig 5(A). A crown-like structure (white arrows) is observed in the adipose tissue of mice injected with *db/db*-derived Ly6C^high^ monocytes (F). (G-I) Quantification of PHK26-labeled *db/db*-derived and *db/+*-derived Ly6C^high^ monocytes in the liver (G), kidney (H), and adipose tissue (I) of recipient *db/db* mice. PKH26 fluorescence was quantified in 16 high-power fields (HPF) per sampley. Results are expressed as the means ± SEM. N = 6 mice/group. *p<0.05. (unpaired t-test).

The liver, adipose tissue, and kidneys play important roles in the regulation of insulin resistance and the development of microvascular complications in obesity and/or diabetes. Therefore, we next asked whether Ly6C^high^ monocytes from *db/db* mice have a greater propensity to infiltrate into these tissues than do the corresponding *db/+* cells. To examine this, Ly6C^high^ monocytes were purified from the bone marrow of *db/+* or *db/db* mice, labeled with the fluorescent dye PKH26, and injected i.v. into recipient *db/db* mice (Fig 5A). One week later, we assessed the number of PKH26+ cells present in the organs, and we found that a significantly higher number of *db/db*-derived than *db/+*-derived Ly6C^high^ monocytes were present in the liver, kidney, and adipose tissue of recipient mice (Fig 6). Moreover, we could detect crown-like structures in the adipose tissue of *db/db* mice injected with *db/db* Ly6C^high^ monocytes, but not *db/+* Ly6C^high^ monocytes (Fig 6E and 6F).

### Exacerbation of diabetes by transfer of Ly6C^high^ monocytes into db/db mice

To clarify the influence of aberrantly activated Ly6C^high^ monocytes on glucose metabolism and homeostasis in diabetic mice, we examined a number of phenotypic changes in *db/db* mice at 4 weeks after transfer of *db/db* or *db/+*-derived Ly6C^high^ monocytes (Fig 5A). Although the body weights of the two groups of mice did not differ at any time point (Fig 5B), serum glucose levels were significantly elevated at 3 and 4 weeks after transfer of *db/db*-derived compared with *db/+*-derived Ly6C^high^ monocytes (Fig 5C and 5D). Additionally, glucose tolerance and insulin sensitivity were both lower in mice that received monocytes from *db/db* mice compared with *db/+* mice (Fig 5E–5H). However, although serum fructosamine and urinary glucose, albumin, and 8-OHdG levels tended to be higher in animals after transfer of *db/db*-derived monocytes than *db/+*-derived monocytes, the differences were not statistically significant (Fig 5I–5L).

## Discussion

In this study, we investigated the potential contribution of aberrantly activated Ly6C^high^ monocytes to the development and phenotype of diabetic and obese mice. Although there were no differences between the ratios of Ly6C^high^ and Ly6C^low^ monocytes in the bone marrow of control *vs* diabetic and obese mice, the latter animals contained more aberrantly activated Ly6C^high^ monocytes expressing higher levels of inflammation-related genes compared with the control mice. The inflammation-related markers were also expressed at higher levels in Ly6C^high^ monocytes from *db/db* mice than from STZ-induced mice, and relatively few genes were upregulated in the cells from HFD-induced mice. This finding suggested that hyperglycemia may have contributed to the aberrant activation of Ly6C^high^ monocytes; however, treatment of *db/db* mice with luseogliflozin had little effect on gene expression, with the exception of *S100a8* and *S100a9*. We attempted to evaluate the effect of insulin resistance alone on inflammatory changes in bone marrow monocytes. Type 2 diabetes is a multifactorial disease with insulin secretion failure and insulin resistance. HFD-treated mice were created as a model for insulin resistance without overt hyperglycemia. An alternative explanation is that the composition or duration of HFD feeding may have been insufficient to promote inflammatory changes in the bone marrow-derived monocytes. However, Ly6C^high^ monocytes from both diabetic and obese mice expressed significantly higher levels of *S100a8* and *S100a9* than control cells. These proteins are known to be involved in NAD(P)H oxidase activation, recruitment of leukocytes [14, 15], and promotion of cytokine and chemokine production. Although changes in *S100a8* and *S100a9* expression could clearly contribute to the aberrant activation of monocytes, clarification of the underlying mechanisms must await further study.

To determine whether the aberrant activation of Ly6C^high^ monocytes in *db/db* and STZ-treated mice was due to hyperglycemia, we treated *db/db* mice with luseogliflozin to prevent glucose resorption, thus lowering blood glucose levels. However, this had no significant effect on inflammation-related gene expression in Ly6C^high^ monocytes from *db/db* mice, making it unlikely that high glucose levels *per se* contributed to the monocyte activation status. Several alternative explanations are possible. One possibility is that as the concentration of DAMPs increases as a result of tissue damage, these changes are not improved in relatively early period. Another possibility is that blood glucose levels declined but obesity accelerated, insulin resistance worsened and blood glucose lowering effect could be canceled. Besides reducing the glucose level, luseogliflozin treatment increased the body weight of *db/db* mice. Our observations are consistent with previous reports in mice [16, 17] treated with SGLT2 inhibitors. They reported that SGLT2 inhibitor might increase appetite and energy intake, which attenuated body weight reduction by urinary calorie loss. Future studies should investigate the effects of sustained and larger reductions in blood glucose, possibly by administration of insulin.

We have developed and validated three pathological models to evaluate the function of bone marrow-derived inflammatory monocytes. Type 2 diabetes is a multifactorial disease with impaired insulin secretion and insulin resistance. Streptozotocin treated mice are the models reflecting insulin secretion failure, and HFD treated mice are the models reflecting insulin resistance. *Db/db* mice are the models with impaired insulin secretion and insulin resistance. In our experimental system, *db/db* mice were positioned as the models most similar to type 2 diabetes. We examined monocyte trafficking to evaluate the relationship between aberrant activation of *db/db*-derived Ly6C^high^ monocytes and chronic inflammation in multiple organs.

Previous work has examined the differentiation of circulating monocytes into tissue-resident macrophages in both lean and obese mice and showed that the CCR2 / MCP-1 system was a factor contributing to monocyte migration to adipocytes and was the main signal controlling the appearance of mobilized macrophages in the liver [18]. We found that *db/db*-derived Ly6C^high^ monocytes showed a higher propensity than their *db/+*-derived counterparts to infiltrate the liver, kidney, and adipose tissue. Moreover, at longer times after cell transfer (2–4 weeks), glucose and insulin tolerance had deteriorated further in animals injected with Ly6C^high^ monocytes from *db/db* mice compared with *db/+* mice. Previous reports have shown that adipocytes, hepatocytes, and other cell types can release chemokines that promote monocyte migration and tissue infiltration [19–22]. The CCR2–MCP-1 axis is thought to play a particularly important role in this regard, but other chemotactic signals can also contribute [18]. Thus, upregulation of the CCR2–MCP-1 axis and other chemotactic signals may have contributed to the increased trafficking of *db/db*-derived compared with *db/+*-derived Ly6C^high^ monocytes. The impaired glucose tolerance of *db/db* mice injected with *db/db*-derived Ly6C^high^ monocytes may also have resulted from secretion of elevated inflammatory cytokine and chemokine levels after differentiation of Ly6C^high^ monocytes into M1 macrophages in the infiltrated tissues. Although IL-1β is known to be involved in the autoimmune process leading to type 1 diabetes, it is also upregulated in the pancreatic islets of diabetic patients and type 2 diabetes animal models, where it causes impaired glucose tolerance [23]. Alternatively, M1 monocytes may have directly infiltrated the pancreatic islets and interacted with β cells via cytokine secretion, further enhancing islet inflammation [24]. Finally, it is possible that the upregulated expression of TLR4 on transferred *db/db*-derived Ly6C^high^ monocytes may have exacerbated glucose intolerance. A recent study showed that TLR4 signaling mediates inflammatory responses in adipose tissue and skeletal muscle leading to HFD-induced insulin resistance [25]. As a result, crown-like structures (CLS) were observed in adipose tissue of mice treated *db/db*-derived monocytes stained with PKH26, but none of them observed in mice treated *db /+*-derived monocytes. CLS were considered to scavenge residual lipid droplets of necrotic adipocytes and reflects adipose inflammation. This result may indicate that abnormally activated *db/db* bone marrow-derived Ly6C^high^ monocytes caused strong inflammation in the infiltrating adipose tissue. In addition, a previous report demonstrated that relatively few macrophages infiltrate into adipose tissue in lean mice compared with obese mice [18], which is consistent with the findings of the present study.

Current experiment has some limitations. First, in transplantation experiments, the effects of inflammatory monocytes from diabetic donor mice on WT recipient mice have not been evaluated. Second, the effects on other systemic organs including the pancreas have not been observed. Therefore, future studies were needed to confirm these detail mechanisms.

In conclusion, our study shows for the first time that (i) Ly6C^high^ monocytes are aberrantly activated in the bone marrow of diabetic mice, (ii) *db/db*-derived Ly6C^high^ monocytes traffic more effectively into the liver, kidneys, and adipose tissue of recipient *db/db* mice than do *db/+*-derived cells, and (iii) recipients of *db/db*-derived Ly6C^high^ monocytes display worse glucose tolerance than do recipients of *db/+*-derived Ly6C^high^ monocytes. Thus, aberrantly activated bone marrow-derived Ly6C^high^ monocytes may contribute to the glucose intolerance of diabetic animal models.

## Supporting information

S1 Table Flow cytometry results of *db/+* and *db/db* mice. (DOC)

S2 Table. Flow cytometry results of vehicle- and STZ-treated mice (DOC)

S3 Table Flow cytometry results of ctrl- and HFD-fed mice. (DOC)

S4 Table Body weight of *db/+* and *db/db* mice. (DOC)

S5 Table Blood glucose levels of *db/+* and *db/db* mice. (DOC)

S6 Table Body weight of vehicle- and STZ-treated mice. (DOC)

S7 Table Blood glucose levels of vehicle- and STZ-treated mice. (DOC)

S8 Table Body weight of ctrl- and HFD-fed mice. (DOC)

S9 Table Blood glucose levels of ctrl- and HFD-fed mice. (DOC)
