# Supplementary material for: Aberrant activation of bone marrow Ly6C high monocytes in diabetic mice contributes to impaired glucose tolerance
Source: PLoS One. 2020 Feb 25;15(2):e0229401. doi: 10.1371/journal.pone.0229401 (PMC7041861; doi:10.1371/journal.pone.0229401)
Supplement: S1 Table — (DOC) [file pone.0229401.s001.doc]

**Supplemental Table 1. Flow cytometry results of *db/+* and *db/db* mice**

| **Fig. #** |  |  |  |  |
| --- | --- | --- | --- | --- |
| **Fig1B** | Mean | SEM | P value | # sample |
| **Ly6Chi(%) db/+** | 89.2 | 2.6 | N.S. | 11 |
| **Ly6Clo(%) db/+** | 10.4 | 2.6 |  | 11 |
| **Ly6Chi(cells) db/+** | 7351.4 | 1296.8 |  | 11 |
| **Ly6Clo(cells) db/+** | 847.6 | 222.9 |  | 11 |
| **Ly6Chi(%) db/db** | 86.6 | 2.9 |  | 11 |
| **Ly6Clo(%) db/db** | 13.0 | 2.9 |  | 11 |
| **Ly6Chi(cells) db/db** | 5259.8 | 721.7 |  | 11 |
| **Ly6Clo(cells) db/db** | 780.8 | 148.8 |  | 11 |
