# Supplementary material for: Aberrant activation of bone marrow Ly6C high monocytes in diabetic mice contributes to impaired glucose tolerance
Source: PLoS One. 2020 Feb 25;15(2):e0229401. doi: 10.1371/journal.pone.0229401 (PMC7041861; doi:10.1371/journal.pone.0229401)
Supplement: S2 Table. Flow cytometry results of vehicle- and STZ-treated mice — (DOC) [file pone.0229401.s002.doc]

**Supplemental Table 2. Flow cytometry results of vehicle- and STZ-treated mice**

| **Fig. #** |  |  |  |  |
| --- | --- | --- | --- | --- |
| **Fig1C** | Mean | SEM | P value | # sample |
| **Ly6Chi(%) Veh** | 92.6 | 2.4 | N.S. | 11 |
| **Ly6Clo(%) Veh** | 7.3 | 2.4 |  | 11 |
| **Ly6Chi(cells) Veh** | 6089.2 | 1538.4 |  | 11 |
| **Ly6Clo(cells) Veh** | 451.6 | 65.6 |  | 11 |
| **Ly6Chi(%) STZ** | 88.1 | 4.0 |  | 11 |
| **Ly6Clo(%) STZ** | 11.6 | 3.9 |  | 11 |
| **Ly6Chi(cells) STZ** | 3707.4 | 1466.4 |  | 11 |
| **Ly6Clo(cells) STZ** | 493.2 | 321.1 |  | 11 |
