# Supplementary material for: Aberrant activation of bone marrow Ly6C high monocytes in diabetic mice contributes to impaired glucose tolerance
Source: PLoS One. 2020 Feb 25;15(2):e0229401. doi: 10.1371/journal.pone.0229401 (PMC7041861; doi:10.1371/journal.pone.0229401)
Supplement: S3 Table — (DOC) [file pone.0229401.s003.doc]

**Supplemental Table 3. Flow cytometry results of ctrl- and HFD-fed mice**

| **Fig. #** |  |  |  |  |
| --- | --- | --- | --- | --- |
| **Fig1D** | Mean | SEM | P value | # sample |
| **Ly6Chi(%) Ctrl** | 85.5 | 5.9 | N.S. | 11 |
| **Ly6Clo(%) Ctrl** | 13.3 | 5.5 |  | 11 |
| **Ly6Chi(cells) Ctrl** | 5803.0 | 2104.0 |  | 11 |
| **Ly6Clo(cells) Ctrl** | 891.2 | 434.2 |  | 11 |
| **Ly6Chi(%) HFD** | 82.5 | 4.0 |  | 11 |
| **Ly6Clo(%) HFD** | 17.4 | 3.9 |  | 11 |
| **Ly6Chi(cells) HFD** | 5296.6 | 1465.1 |  | 11 |
| **Ly6Clo(cells) HFD** | 1179.2 | 313.8 |  | 11 |
