# Supplementary material for: Aberrant activation of bone marrow Ly6C high monocytes in diabetic mice contributes to impaired glucose tolerance
Source: PLoS One. 2020 Feb 25;15(2):e0229401. doi: 10.1371/journal.pone.0229401 (PMC7041861; doi:10.1371/journal.pone.0229401)
Supplement: S4 Table — (DOC) [file pone.0229401.s004.doc]

**Supplemental Table 4. Body weight of *db/+* and *db/db* mice**

| **Fig. #** |  | | | | |
| --- | --- | --- | --- | --- | --- |
| **Fig2A** |  | | | | |
| **weeks** | 8 | 10 | 12 | 14 | 16 |
| **Mean db/+** | 24.14545 | 27.39091 | 28.20909 | 29.50455 | 30.8 |
| **Mean db/db** | 35.17091 | 46.14545 | 47.19091 | 50.21818 | 53.24545 |
| **SE db/+** | 0.746142 | 0.926773 | 1.221847 | 0.691901 | 0.507937 |
| **SE db/db** | 0.733286 | 1.413764 | 1.484894 | 1.179888 | 1.593966 |
| **P value** | P<0.01 | | | | |
