# Supplementary material for: Aberrant activation of bone marrow Ly6C high monocytes in diabetic mice contributes to impaired glucose tolerance
Source: PLoS One. 2020 Feb 25;15(2):e0229401. doi: 10.1371/journal.pone.0229401 (PMC7041861; doi:10.1371/journal.pone.0229401)
Supplement: S5 Table — (DOC) [file pone.0229401.s005.doc]

**Supplemental Table 5. Blood glucose levels of *db/+* and *db/db* mice**

| **Fig. #** |  | | | | |
| --- | --- | --- | --- | --- | --- |
| **Fig2B** |  | | | | |
| **weeks** | 8 | 10 | 12 | 14 | 16 |
| **Mean db/+** | 132.3636 | 127.4545 | 154 | 153.0455 | 152.0909 |
| **Mean db/db** | 444.0909 | 537.8182 | 495 | 563.0455 | 631.0909 |
| **SE db/+** | 22.22284 | 12.37226 | 20.41568 | 21.97778 | 34.28252 |
| **SE db/db** | 77.4396 | 77.41553 | 42.78317 | 75.06912 | 114.8046 |
| **P value** | P<0.01 | | | | |
