# Supplementary material for: Aberrant activation of bone marrow Ly6C high monocytes in diabetic mice contributes to impaired glucose tolerance
Source: PLoS One. 2020 Feb 25;15(2):e0229401. doi: 10.1371/journal.pone.0229401 (PMC7041861; doi:10.1371/journal.pone.0229401)
Supplement: S6 Table — (DOC) [file pone.0229401.s006.doc]

**Supplemental Table 6. Body weight of vehicle- and STZ-treated mice**

| **Fig. #** |  | | | | | |
| --- | --- | --- | --- | --- | --- | --- |
| **Fig2C** |  | | | | | |
| **weeks** | 8 | | 10 | 12 | 14 | 16 |
| **Mean Veh** | 24.09167 | | 27.88333 | 30.75 | 32.46667 | 35.30833 |
| **Mean STZ** | 24.78333 | | 23.825 | 25.11667 | 23.95833 | 24.03333 |
| **SE Veh** | 1.514451 | | 1.297433 | 0.944361 | 1.487728 | 1.630091 |
| **SE STZ** | 2.262273 | | 1.782809 | 1.513475 | 2.022806 | 1.825244 |
| **P value** | N.S. | P<0.01 | | | | |
