# Supplementary material for: Aberrant activation of bone marrow Ly6C high monocytes in diabetic mice contributes to impaired glucose tolerance
Source: PLoS One. 2020 Feb 25;15(2):e0229401. doi: 10.1371/journal.pone.0229401 (PMC7041861; doi:10.1371/journal.pone.0229401)
Supplement: S7 Table — (DOC) [file pone.0229401.s007.doc]

**Supplemental Table 7. Blood glucose levels of vehicle- and STZ-treated mice**

| **Fig. #** |  | | | | | |
| --- | --- | --- | --- | --- | --- | --- |
| **Fig2D** |  | | | | | |
| **weeks** | 8 | | 10 | 12 | 14 | 16 |
| **Mean Veh** | 145.5833 | | 157.3333 | 153 | 168.5833 | 171.0833 |
| **Mean STZ** | 143.4167 | | 593.5 | 540.4167 | 561 | 582 |
| **SE Veh** | 3.605551 | | 24.41854 | 26.16845 | 20.79773 | 25.4896 |
| **SE STZ** | 3.605551 | | 24.02823 | 79.01611 | 63.90541 | 96.40351 |
| **P value** | N.S. | P<0.01 | | | | |
