# Supplementary material for: Aberrant activation of bone marrow Ly6C high monocytes in diabetic mice contributes to impaired glucose tolerance
Source: PLoS One. 2020 Feb 25;15(2):e0229401. doi: 10.1371/journal.pone.0229401 (PMC7041861; doi:10.1371/journal.pone.0229401)
Supplement: S8 Table — (DOC) [file pone.0229401.s008.doc]

**Supplemental Table 8. Body weight of ctrl- and HFD-fed mice**

| **Fig. #** |  | | | | | |
| --- | --- | --- | --- | --- | --- | --- |
| **Fig2E** |  | | | | | |
| **weeks** | 8 | | 12 | 16 | 20 | 24 |
| **Mean Ctrl** | 25.59167 | | 31.95833 | 35.35833 | 36.45 | 39.5 |
| **Mean HFD** | 26.06667 | | 42.54167 | 47.875 | 50.225 | 53.90833 |
| **SE Ctrl** | 0.830617 | | 1.978732 | 2.518823 | 2.515588 | 1.638736 |
| **SE HFD** | 1.360036 | | 2.063738 | 2.189697 | 1.737357 | 2.860533 |
| **P value** | N.S. | P<0.01 | | | | |
