# Supplementary material for: Aberrant activation of bone marrow Ly6C high monocytes in diabetic mice contributes to impaired glucose tolerance
Source: PLoS One. 2020 Feb 25;15(2):e0229401. doi: 10.1371/journal.pone.0229401 (PMC7041861; doi:10.1371/journal.pone.0229401)
Supplement: S9 Table — (DOC) [file pone.0229401.s009.doc]

**Supplemental Table 9. Blood glucose levels of ctrl- and HFD-fed mice**

| **Fig. #** |  | | | | |
| --- | --- | --- | --- | --- | --- |
| **Fig2F** |  | | | | |
| **weeks** | 8 | 12 | 16 | 20 | 24 |
| **Mean Ctrl** | 171.8333 | 159 | 148.0833 | 166.8333 | 159.5 |
| **Mean HFD** | 183.25 | 172.0833 | 173.0833 | 185.5 | 186.8333 |
| **SE Ctrl** | 3.605551 | 19.33359 | 18.21089 | 23.99416 | 27.15221 |
| **SE HFD** | 3.615692 | 22.37338 | 47.01152 | 80.70762 | 28.92467 |
| **P value** | N.S. | | | | |
